# Multi-centre normative brain mapping of intracranial EEG lifespan patterns in the human brain

**DOI:** 10.1007/s00429-025-02988-4

**Published:** 2025-08-21

**Authors:** Heather Woodhouse, Gerard Hall, Callum Simpson, Csaba Kozma, Frances Turner, Gabrielle M. Schroeder, Beate Diehl, John S. Duncan, Jiajie Mo, Kai Zhang, Aswin Chari, Martin Tisdall, Friederike Moeller, Chris Petkov, Matthew A. Howard, George M. Ibrahim, Elizabeth Donner, Nebras M. Warsi, Raheel Ahmed, Peter N. Taylor, Yujiang Wang

**Affiliations:** 1 CNNP Lab (www.cnnp-lab.com), Interdisciplinary Computing and Complex BioSystems Group, School of Computing, Newcastle University, Newcastle upon Tyne, UK; 2 Faculty of Medical Sciences, Newcastle University, Newcastle upon Tyne, UK; 3 UCL Queen Square Institute of Neurology, London, UK; 4 Beijing Tiantan Hospital, Beijing, China; 5 Great Ormond Street Hospital for Children, London, UK; 6 University of Iowa Hospitals and Clinics, Iowa City, IA USA; 7 The Hospital for Sick Children, Toronto, Canada; 8 University of Wisconsin-Madison, Madison, WI USA

**Keywords:** Intracranial EEG, Normative modelling, Lifespan patterns, Sex differences, Hospital effects, Band power

## Abstract

Understanding healthy human brain function is crucial to identify and map pathological tissue within it. Whilst previous studies have mapped intracranial EEG (icEEG) from non-epileptogenic brain regions, they often neglect age and sex effects. Further, they are limited by small sample sizes due to the modality’s invasive nature. This study substantially expands the subject pool compared to existing literature, to create a multi-centre, normative map of brain activity which considers the effects of age, sex and recording hospital. Using interictal icEEG recordings from $n = 502$ subjects across 15 centres, we constructed a normative map of non-pathological brain activity by regressing age and sex on relative band power in five frequency bands. A linear mixed model was implemented to account for the hospital effect. Variable importance was assessed using standard statistical measures, and regression coefficients (and their standard errors) were analysed at both whole-brain and regional scales. Recording hospital significantly impacted normative icEEG maps in all frequency bands, and age was a more influential predictor of band power than sex. The age effect varied by frequency band, but no spatial patterns were observed at the region-specific level. Certainty about regression coefficients was also frequency band specific and moderately impacted by sample size. The concept of a normative map is well-established in neuroscience research and particularly relevant to the icEEG modality, which does not allow healthy control baselines. Our key results regarding the hospital site and age effect guide future work utilising normative maps in icEEG.

**Supplementary Information:**

The online version contains supplementary material available at 10.1007/s00429-025-02988-4.

## Introduction

Age and sex are important factors which are known to influence brain activity. Understanding *how* these variables affect the brain is important for both clinical applications and academic research. Using scalp EEG recordings in children and adolescents, past studies found that age has a negative relationship with relative power in slower frequency bands, namely $\delta$ and $\theta$, but a positive relationship with faster ones ($\alpha$, $\beta$) (Gasser et al. 1988; Clarke et al. 2001). Both MEG and scalp EEG studies report some sex differences in the same age range, with males tending to have more $\alpha$-power (Ott et al. 2021; Clarke et al. 2001). A study in MEG across the whole lifespan found similar frequency band-specific relationships between age and power, whilst a young adult study in scalp EEG reported various sex differences during the resting state (Cave and Barry 2021; Gómez et al. 2013). In summary, whilst consistent effects across the lifespan have been reported, a conclusive map does not currently exist for electrical brain activity as it does for structural neuroimaging (Bethlehem et al. 2022).

Although much work has been done to assess the impact of age and sex on MRI, MEG and scalp EEG, to our knowledge intracranial EEG (icEEG) has not yet been investigated in this context, most likely because its invasive nature precludes data collection from healthy controls (Gomez et al. 2017; Hinault et al. 2022; Cam-CAN et al. 2014; Coffey et al. 1998). To overcome this issue, researchers have collected icEEG recordings from individuals with epilepsy, from brain regions that were later deemed not pathological and not epileptogenic. By combining these recordings over many individuals, a map of normative brain activity has been proposed (Groppe et al. 2013; Frauscher et al. 2018a; Taylor et al. 2022; von Ellenrieder et al. 2020). Whilst there has been exciting research into normative maps using icEEG and their potential for epilepsy research, the effect of age and sex on icEEG has not been determined (Taylor et al. 2022; Frauscher et al. 2018a; Kalamangalam et al. 2020; Wang et al. 2023; Bernabei et al. 2022; Betzel et al. 2019).

To analyse this effect thoroughly, this investigation utilises a large dataset, encompassing multiple international hospitals. This is similar to Bethlehem et al. (2022) which leverages MRI scans from various studies globally to create brain charts for the human lifespan—our study seeks to help establish such methods in the icEEG literature. Importantly, we will perform our analysis on the largest multi-centre normative icEEG dataset to date ($n=502$ subjects), accounting for hospital site effects in data by using mixed-effect modelling. Since we amalgamate data across hospitals, we will also examine whether the recording hospital has any systematic effect on results, helping us to account for a multitude of possible differences between them. Following this, we aim to uncover and discuss the relationships, if any, between band power extracted from icEEG recordings, and the variables age and sex.

Ultimately, we highlight the need to account for the heterogeneity of icEEG data. We seek to fill a gap in the literature regarding the effect of two standard covariates—age and sex—on band power values extracted from icEEG and modelled in a normative setting. We also expand on previous sample sizes and properly consider hospital variability. Such research is necessary, as an understanding of ageing patterns, sex differences and hospital variation in this modality will conceivably aid in the identification of pathological activity through deviations from ‘normal’ trends.

## Methods

A portion of the methodology used here (Sects. 2.2 and 2.3) has been outlined in more detail in our data protocol (Woodhouse et al. 2025). Nevertheless, we outline all steps for clarity.

### Subjects

Our analysis involved 502 individuals with epilepsy undergoing presurgical evaluation with icEEG to localise the seizure onset zone. Data was collected from Beijing Tiantan Hospital, Great Ormond Street Hospital, University of Iowa Hospital, SickKids, University College London Hospital and the University of Wisconsin-Madison. We also used nine hospitals from the publicly available RAM dataset (https://memory.psych.upenn.edu/RAM), bringing our total number of contributing hospital sites to 15.

**Fig. 1:**
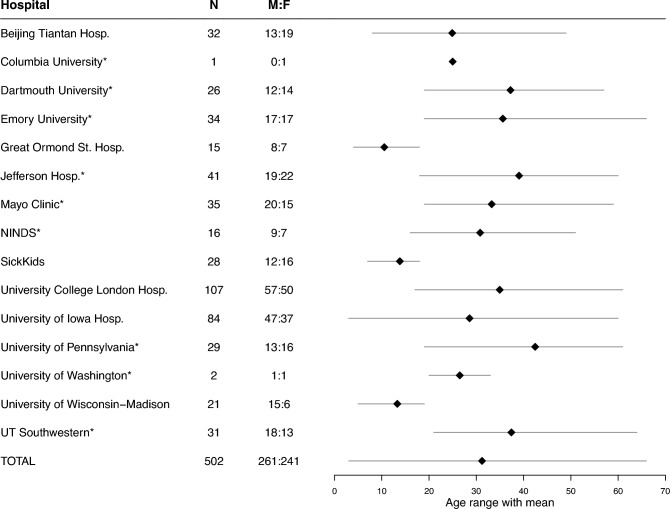
The number of subjects from each participating hospital, along with age and sex distributions. Hospitals from the RAM cohort are highlighted with an *

Anonymised icEEG recordings were analysed following approval of the Newcastle University Ethics Committee (reference number 23973/2022). Both grid and depth electrode recordings were included. All subjects were recorded using a referential montage. A summary of our final cohort is provided in Fig. 1.

### Electrode localisation

For every participant, electrode contacts were localised to regions of interest (ROIs) according to a predefined parcellation, using the “Lausanne scale 36" atlas, with 82 ROIs (Hagmann et al. 2008). The Lausanne atlas has been used previously for normative intracranial analysis (Betzel et al. 2019; Taylor et al. 2022). The methods for localising the electrode contacts to brain regions have also been described previously (Taylor et al. 2022; Wang et al. 2020; Hamilton et al. 2017; Wang et al. 2023).

As this is retrospective data, there was a range of variables which differed across sites that we could not control for (also see Discussion). For example, different hospital sites provided different levels of data, so our methods for localising electrode contacts to ROIs varied between them. Hospitals either provided contact locations in MNI space, or provided native space imaging and co-localised contacts. In the first case, we assigned electrodes to one of 82 regions from the Lausanne scale 36 atlas (Hagmann et al. 2008). We used FreeSurfer to generate volumetric parcellations of an MNI space template brain (Fischl 2012; Hagmann et al. 2008). Each electrode contact was assigned to the closest grey matter volumetric region within 5 mm. If the closest grey matter region was >5 mm away then the contact was excluded from further analysis. For the latter case, a similar technique was used, but applied in native space using the subject’s own parcellated pre-operative MRI.

To ensure our findings were robust to parcellation choice, we confirmed that key results held using a finer-grained parcellation (Supplementary S3).

### icEEG processing

#### Segment selection

For each subject, we extracted a 70-second interictal icEEG segment from a period of relaxed wakefulness, at least two hours away from any detected ictal events.

The recordings from the RAM cohort (representing 43% of all subjects), were obtained in the preparatory phase, several minutes before a memory task, confirming these subjects were awake. The remaining data collaborators sent either (a) interictal recordings from periods of relaxed wakefulness only or (b) long-term recordings. In the latter case, the state of vigilance and interictal criteria were fulfilled using clinical annotations, reports and time of day. For example, where there is a clear label in the EEG of ‘awake—resting’ we selected our segment around this label. Nevertheless, we cannot guarantee the vigilance state for each subject, and when working with retrospective multi-site data, this is a clear limitation, which we also highlight in Discussion.

The raw signals and the power spectral densities were visually and algorithmically inspected for spikes, artefacts and faulty channels (see Supplementary S1 for details). Using clinical reports (if available) we excluded any contacts that were: within known lesions, within the seizure onset zone, or subsequently resected. This ensured that at the subject level, we only retained channels thought to be non-pathological in terms of both location in the brain and the signal produced. If unavailable in clinical reports, information on resected contacts could also be obtained by drawing masks using post- and pre-operative scans (where available), as in Taylor et al. (2022) and Wang et al. (2020). Supplementary S2 details the exclusionary information available for each hospital (whether via clinical reports or resection masks).

#### Signal processing

All segments were bandpass filtered between 0.5–80 Hz and downsampled to 200 Hz with an anti-alias filter. A common average reference was applied to all recordings and the power spectral density was calculated using Welch’s method with a 2-second window and 1-second overlap. The average band power was then calculated in five frequency bands: $\delta$ (1–4 Hz), $\theta$ (4–8 Hz), $\alpha$ (8–13 Hz), $\beta$ (13–30 Hz) and $\gamma$ (30–77.5 Hz).

In the $\gamma$-band, data between 47.5$-$52.5 Hz and 57.5$-$62.5 Hz were excluded from all hospital sites, to avoid any power line noise. The $\gamma$-band was also capped at 77.5 Hz due to 80 Hz noise in the RAM cohort. Band power estimates were $\hbox {log}_{10}$ transformed and normalised to sum to one in each contact (L1-normalised). These final values are used throughout results to represent relative band power, denoted RBP($\cdot$).

### Normative data table creation

At this stage, the electrode contacts from each subject have been assigned to a single, nearest ROI and the RBP($\cdot$) in five frequency bands has been computed. If multiple contacts from one subject were assigned to the same ROI, then the RBP($\cdot$) values in each frequency band were averaged across contacts to obtain single values of RBP($\cdot$) per region per subject.

We excluded six sub-cortical regions due to no, or a very low number of samples: pallidum, thalamus and accumbens area in both hemispheres, reducing our total number of ROIs from 82 to 76. All cortical regions were retained.

Previous work has demonstrated a left/right symmetry in RBP($\cdot$) in EEG, MEG, and icEEG (Taylor et al. 2022; Janiukstyte et al. 2023; Owen et al. 2023b). Hence, to maximise the number of subjects in each region, we created a ‘mirrored’ version of our data table, in which, the data from homologous regions were combined (e.g., the left amygdala and the right amygdala). In the case of bilateral implantation of symmetric regions, we ensured individuals only had one value of RBP($\cdot$) per ROI and frequency band. This mirrored data table only comprised 38 regions but had markedly more subjects in each. Throughout the results, it will be made clear whether the mirrored (regional), or original (whole-brain), normative data table is in use at any given time. We confirm the validity of this mirroring in Supplementary S4 by repeating one of our key results using the original data.

As demonstrated in Fig. 1, all subjects had age and sex reported. However, due to the size and multi-centre nature of our data, no other variables of interest were consistently available across subjects. This prevented further investigation of their effect on spectral properties, without reducing the sample size. Taking a subset of the data, Supplementary S5 demonstrates that our normative values are not related to a subject’s age of epilepsy onset, a feature representing pathology. Unfortunately, we do not have the data to check other disorder-specific features, such as drug levels or epilepsy classification. Supplementary S2 provides details on which clinical variables were reported for each hospital.

In summary, our final normative data table included a unique subject identifier, their age and sex, their originating hospital, and, for the regions in which they had electrode implantation only, their RBP($\cdot$) in five frequency bands averaged across contacts where necessary.

### Statistical modelling and testing

The final step was to fit a suitable model to the normative data to examine the effect of age, sex and hospital site on relative band power. Visual inspection of scatter plots of the RBP($\cdot$) values against age in each frequency band (at the whole-brain level) showed no evidence of a non-linear relationship (see Supplementary S6). Further, the effect of having multiple recording hospitals had to be considered, especially as some hospitals supplied only paediatric, or only adult recordings. Therefore, we implemented a linear mixed model (LMM) in each frequency band, specifically a random intercept model, with the originating hospital site as a random effect. Our cohort has a wide range of subjects per hospital (Fig. 1), but one of the strengths of mixed modelling is the ability to handle unequal group sizes (West et al. 2014).

Possible fixed effects were age and sex, meaning four LMMs were under consideration in each frequency band:

$$\begin{aligned}{\textbf {Null:}} \; & RBP(\cdot ) \sim (1|Hospital) \\{\textbf {Age:}} \; & RBP(\cdot ) \sim Age +(1|Hospital) \\{\textbf {Sex:}} \; & RBP(\cdot ) \sim Sex +(1|Hospital) \\{\textbf {Full:}} \; & RBP(\cdot ) \sim Age + Sex +(1|Hospital) \end{aligned}$$

An interaction term between age and sex was also considered but deemed unnecessary (Supplementary S7). Model fitting could be performed at either the regional or whole-brain level. Taking the latter, we calculated several model evaluation statistics to determine the optimal fixed effect structure of the LMM in each frequency band. Since we worked at the whole-brain level, the original data table was used. The statistics under consideration were the AIC and BIC for each model, 95% profiled confidence intervals for the regression coefficients of fixed effects in all but the null model (which has none) and the likelihood ratio test *p*-values for every pair of models which differed by one variable.

In four frequency bands, each metric was in agreement regarding the optimal LMM, so no further tests were required. The preferred models were the age model in the $\delta$ and $\beta$ bands; the sex model in $\gamma$ and the full model in $\theta$. The $\alpha$ band demonstrated some uncertainty between the age model and the full model. Since both models perform similarly, we retain the simpler age model as the optimal choice in the $\alpha$ band.

**Fig. 2:**
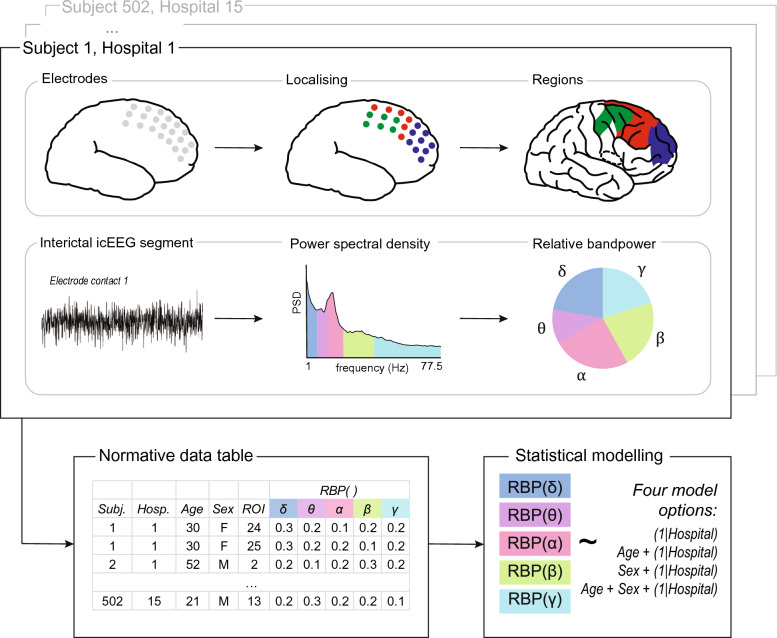
A visual representation of our methods: electrode localisation (top) and signal processing following segment selection (middle), for each subject. Subjects were combined to create our normative data table, to which we applied our statistical models (bottom). All components show dummy data for example purposes only. Brain plots from Scholtens et al. (2021)

**Fig. 3:**
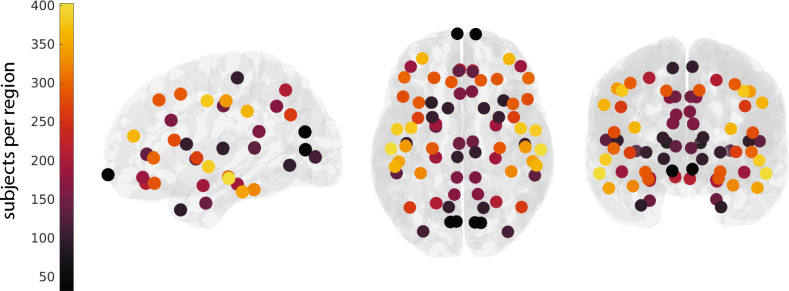
A visual representation of the number of subjects recorded in each ROI in the normative, mirrored data frame. Bright yellows and oranges indicate a higher number of subjects per region, whilst deep purples and black indicate the opposite. Note, lateral regions are notably more densely populated than medial regions. For the axial and coronal views (central and right plots), data from symmetric regions were mirrored, i.e., sample sizes are reflected across the midline to provide a whole-brain visualisation

Hence, the optimal LMM was frequency band-specific in our cohort, and so we report frequency band-specific effects throughout our results. We have provided a brief visual overview of our methods (Fig. 2), and a visualisation of the number of subjects per region in the mirrored normative dataset (Fig. 3). In the results section, we explore both random (hospital site) and fixed (age, sex) effects. Specifically, we investigate if the variables explain RBP($\cdot$) variation, and examine how they might influence it. We also consider the impact of regional sample size.

## Results

Results “Hospital site effects impacted icEEG normative maps”, “Age and sex explained only some of the variation in relative band power” and “Age was more important than sex for predicting relative band power” examine LMMs at the whole-brain level, using the original data. Subsequent results additionally consider the regional-level analysis, and therefore incorporate the mirrored data, to determine if any spatial variations are present in our findings.

### Hospital site effects impacted icEEG normative maps

Focusing first on the random effect structure (recording hospital), we found that in our cohort and model, the hospital site effect was much more powerful in explaining RBP($\cdot$) differences at the whole-brain level than any fixed effect structure, in almost all cases. Table 1 quantifies this, showing marginal $R^2$ values ($R^2_m$) and intraclass correlation coefficients (*ICC*) as percentages for the full model, the age model and the sex model. The $R^2_m$ represents the proportion of variation in RBP($\cdot$) explained by the fixed effect(s) of that model. The *ICC* represents the proportion of the variance explained by the recording hospitals. The optimal LMM as per Sect. 2.5 has been highlighted for each frequency band.

Note, the $R_m^2$ values of the two single fixed effect models do not sum exactly to the $R^2_m$ of the full model, because the denominator in the calculation of $R_m^2$ includes the residual variance and therefore changes with each model.

**Table 1:**
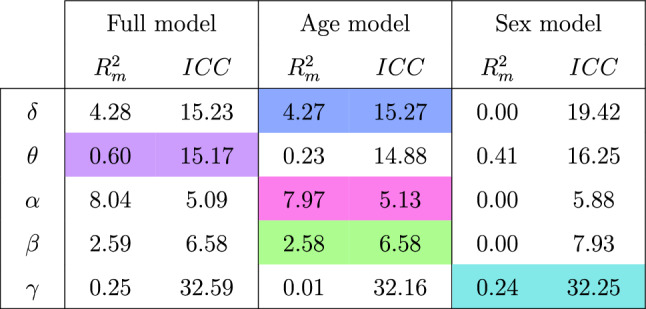
$R^2_m$ and *ICC* values (measured in %) for the full model, the age model and the sex model

$R^2_m$ represents the proportion of variation in RBP($\cdot$) explained by the fixed effect(s) of that model. The *ICC* represents the proportion of the variance explained by the grouping structure, namely recording hospitals. The optimal covariate subset, as determined by a standard model selection process, is highlighted for each frequency band

There is striking variation in the magnitude of the hospital site effect on RBP($\cdot$) across signal properties. However the *ICC*s consistently exceed 5%, indicating that, in our cohort, at least a twentieth of the variation in RBP($\cdot$) was explained by the random effect structure. This proportion sometimes reached as high as 30%, attributing that recording hospital impacted RBP($\cdot$) in all frequency bands. Further, in all bands except $\alpha$, the *ICC* was consistently larger than the $R^2_m$ values across models, with this difference being most notable in the $\theta$ and $\gamma$ bands.

**Fig. 4:**
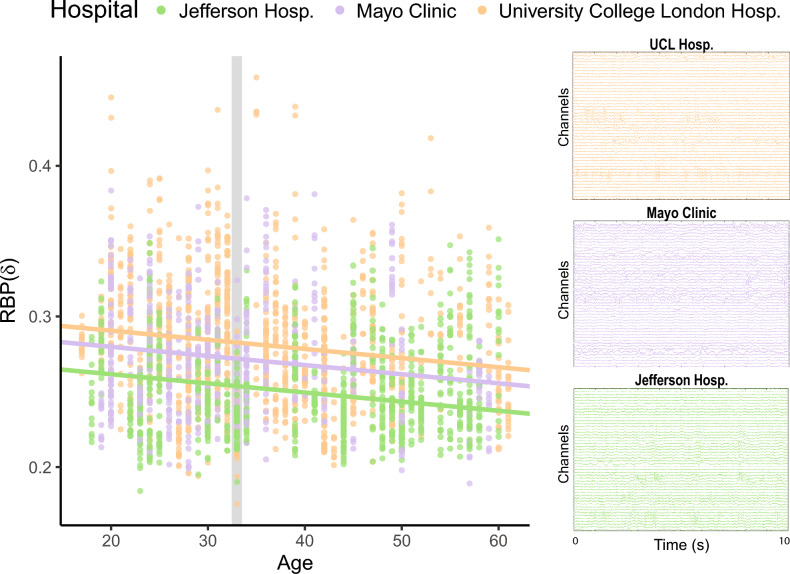
Scatterplot of RBP($\delta$) against age for thee well-populated hospitals. The age model was implemented for all hospitals, then the age slope and intercepts for Jefferson Hospital, Mayo Clinic and University College London Hospital were extracted and plotted. For three similar individuals, men of age 33, the first 50 channels and 10 s of their 70-second preprocessed icEEG segments have been provided. The grey bar indicates where the segments from the 33-year-old men lie within this subset of the data

To visualise the effect of the originating hospital on RBP($\cdot$), we fit the age model to all data, then selected three well-populated hospitals with similar age ranges and plotted RBP($\delta$) against age, along with the model fit in Fig. 4. Additionally, we identified a 33-year-old male from each of the three hospitals and have provided the first 10 s of their processed 70-second icEEG segment. The three subjects had variable numbers of channels, so only the first 50 channels are displayed for comparative purposes. Figure 4 demonstrates that while individuals’ icEEG segments may appear similar, the originating hospital introduces underlying differences to relative band power properties.

Visualisations of the intercept offset for all five frequency bands and all hospitals can be found in Supplementary S8. To demonstrate the quality of the data, Supplementary S9 examines the icEEG for the three 33-year-old male subjects in more detail.

### Age and sex explained only some of the variation in relative band power

We next examined our fixed effects at the whole-brain level ($R^2_m$). Neither age nor sex explained a substantial portion of the variance in the RBP($\cdot$), in any frequency band (Table 1). The largest effect was found in the $\alpha$-band, where $R^2_m$ was highest at 8%, which in contrast, is similar to the minimum *ICC* values at around 5%.

Across fixed effects and frequency bands, all $R^2_m$ values were below 10%, with over half being less than 1%. This suggests that other factors significantly affect RBP($\cdot$) on the whole-brain level, which are not considered here.

### Age was more important than sex for predicting relative band power

Comparing $R^2_m$ for the age and sex models revealed which of the two was more valuable when predicting RBP($\cdot$). All bands which achieve $R^2_m>1\%$ do so only when age is included in the model (Table 1). In fact, sex consistently accounted for a negligible portion of the variance in the response, with $R^2_m$ values below 0.5%. This statement holds even in $\theta$ and $\gamma$, with sex retained in the optimal LMM, confirming sex was not a significant predictor in this cohort. In contrast, $R^2_m$ values for age fluctuated notably across frequency bands, being higher in $\delta$, $\alpha$ and $\beta$ ($R^2_m$ range of $7.96\%$ and $0.41\%$ for age and sex models respectively).

**Fig. 5:**
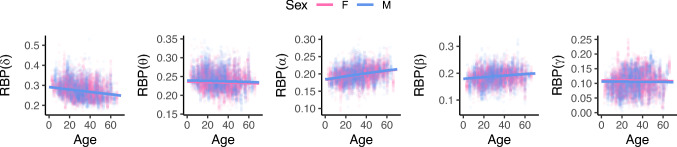
Scatterplots of RBP($\cdot$) against age in every frequency band, using data at the whole-brain level. The full model has been implemented and the male/female regression lines have been plotted

To validate the lack of sex effect in this cohort, we plotted RBP($\cdot$) against age for all frequency bands at the whole brain level. We then fit the full model and plotted the male/female regression lines. Figure 5 demonstrates visually that sex is not a significant predictor in this cohort, with the differing sex lines being indistinguishable in almost all bands.

Together, Table 1 and Fig. 5 emphasize that the influence of sex was minor, and age was the more important predictor of RBP($\cdot$) in our cohort. Hence, subsequent analysis focuses on the age model for all frequency bands; however, we exercise caution when interpreting results in the $\theta$ and $\gamma$ bands, which have a weak relationship with both fixed effects.

### The effect of age on band power was spatially uniform and frequency band dependent

With the focus shifted to the age model alone, our goal was to understand *how* age impacted RBP($\cdot$).

**Fig. 6:**
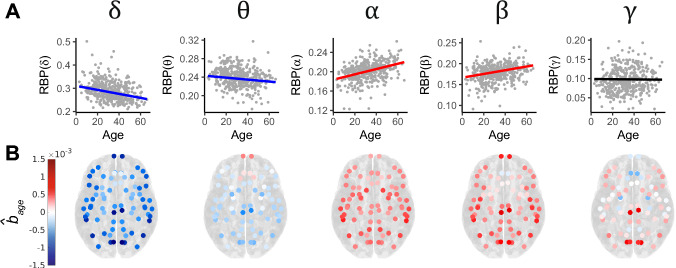
**A** Visualisations of the age model in each frequency band in the most densely populated region, the middle temporal. Using the 95% confidence intervals for ${\hat{b}}_{age}$ in the region, blue regression lines indicate a negative relationship between RBP($\cdot$) and age, whilst red regression lines indicate a positive one and black indicates no relationship. **B** Values of ${\hat{b}}_{age}$ from the age model at the region-level. Values are shown for each ROI and each frequency band of interest. The colour scale is symmetric and fixed across frequency bands with blue representing negative regression coefficients and red representing positive ones. Data from symmetric regions were mirrored, i.e. results are reflected across the midline to provide a whole-brain visualisation

Taking the most densely populated ROI (the middle temporal region, $n=403$), the age model was implemented for RBP($\cdot$) in each frequency band and regression coefficients for age (denoted ${\hat{b}}_{age}$) were extracted. A visualisation of model fit in this region is provided in Fig. 6A, where blue coloured lines indicate that RBP($\cdot$) decreased with age, red lines indicate an increase with age and black lines indicate no relationship (based on 95% confidence intervals for ${\hat{b}}_{age}$). This method can be extended to all regions, extracting ${\hat{b}}_{age}$ for each frequency band and region. The ${\hat{b}}_{age}$ values can then be visualised on the brain using a hot/cold colour scale, indicating increasing/decreasing RBP($\cdot$) with age (Fig. 6B). Finally, disregarding regional information, at the whole-brain level, the age model was implemented in each frequency band and regression coefficients were extracted, along with their 95% confidence intervals (Table 2). Looking at both whole-brain and ROI-level results allowed us to determine if the results had any spatial variation.

Opposing relationships between RBP($\cdot$) and age were observed at both the region-specific and whole-brain levels, with decreasing trends in $\delta$ and $\theta$ frequencies, increasing trends in the $\alpha$ and $\beta$ bands and weaker results found for the $\gamma$ band.

Figure 6B reveals no discernible spatial gradient in any frequency band, but a clear switch in the sign of ${\hat{b}}_{age}$ occurs between $\theta$ and $\alpha$, from negative to positive. The $\theta$ and $\gamma$ trends were notably weaker, presenting values of ${\hat{b}}_{age}$ which are closer to zero, along with some regions deviating from the overarching trends. This is consistent with Table 1 showing that for these two bands, the relationship between age and RBP($\cdot$) is weak, when compared with $\delta$, $\alpha$ and $\beta$.

**Table 2:** Values of ${\hat{b}}_{age}$ along with 95% confidence intervals for each frequency band

$\delta$	$\theta$	$\alpha$	$\beta$	$\gamma$
– 6.1 × 10^4^	– 0.8 × 10^4^	4.1 × 10^4^	2.9 × 10^4^	– 0.2 × 10^4^
(– 6.7,– 5.4) × 10^4^	(– 1.2,– 0.4) × 10^4^	(3.8, 4.5) × 10^4^	(2.4, 3.3) × 10^4^	(– 0.7, 0.3) × 10^4^

Values are rounded to 5 decimal places and were calculated by applying the age model at the whole-brain level

On the whole-brain level, 95% confidence intervals on ${\hat{b}}_{age}$ reinforced previous results. Table 2 provides no evidence for an age-RBP($\gamma$) relationship and $\theta$’s confidence interval is relatively near zero compared with the remaining three bands. These confidence intervals support the existence of a negative relationship between RBP($\delta$) and age, along with a positive one between RBP($\alpha$) and RBP($\beta$), and age. Hence, the effect of age on RBP($\cdot$) was undoubtedly frequency band-specific in this cohort.

The subset of subjects varies between ROIs in the regional analysis; however, the age distribution in each region did not drive any differences between them (Supplementary S10). The trends in Fig. 6B persist in a finer-grained parcellation, and when using the original data in which hemispheres were not mirrored (Supplementary S3 and S4).

### Certainty about age effect was frequency band specific and impacted by sample size

**Fig. 7:**
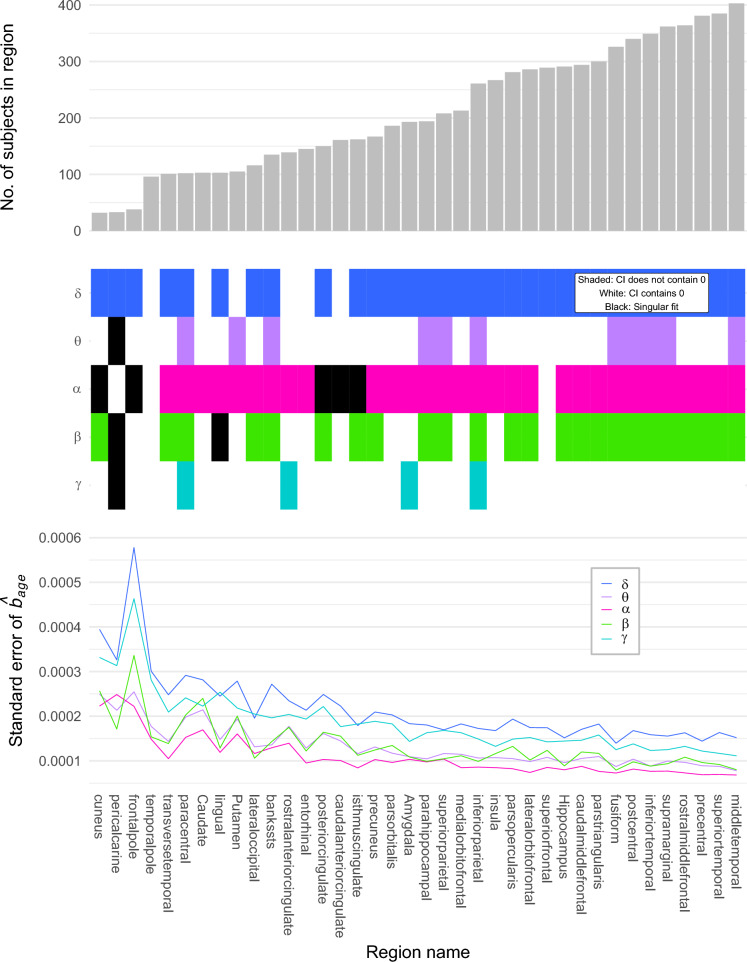
Summaries for each ROI under consideration. The *x*-axis displays the region name and is ordered by the number of subjects per region. **A** number of subjects. **B** a binary indicator of whether the 95% confidence interval for ${\hat{b}}_{age}$ contains 0 in each frequency band. Singular fits given in black. **C**) standard errors of ${\hat{b}}_{age}$ in each frequency band

Despite substantial data collection efforts, when analysing region-level models there was an impact of low sample size. Figure 7 displays region-level summaries in every frequency band: the standard error for ${\hat{b}}_{age}$, the number of subjects in the region, and a binary indicator of whether or not the 95% confidence interval for ${\hat{b}}_{age}$ contains 0. It also highlights any regions where the model produced a singular fit.

As a side note, extreme values in Fig. 6B co-localise to low sampled regions seen in Fig. 7 and Fig. 3, e.g., the frontal pole stands out from the general trend in $\theta$ and only has $n=38$, highlighting the importance of sample size.

The standard errors revealed that smaller sample sizes can lead to regression coefficient standard errors more than double those of the highly implanted ROIs. However, there appeared to be a threshold around $n=150$, beyond which the ${\hat{b}}_{age}$ standard error lines plateaued, suggesting a lower limit of *n*, beyond which we can be confident in our findings.

The $\delta$ band displayed the highest regression coefficient standard errors almost consistently across ROIs, followed by $\gamma$ with slightly lower values. Comparatively, $\alpha$, $\beta$ and $\theta$ bands exhibited lower ${\hat{b}}_{age}$ standard errors. In regions with approximately $n>200$, the $SE({\hat{b}}_{age})$ values dropped below 0.0002. For $\alpha$, these same regions consistently showed a regression coefficient standard error of half the size, supporting the positive relationship between RBP($\alpha$) and age.

ROI-level confidence intervals also demonstrated that ${\hat{b}}_{age}$ standard errors varied with frequency band and sample size. Figure 7B shows that in $\theta$ and $\gamma$ bands, regions are dominated by confidence intervals for ${\hat{b}}_{age}$ that contain 0, whereas the converse is true for $\delta$, $\alpha$ and $\beta$.

With the exception of the $\theta$-band, these findings became robust in ROIs with $n \ge 291$. This suggests that equal and large sample sizes in all ROIs may lead to more uniform results and provides further confidence in the relationships we have identified between age and $\delta$, $\alpha$ and $\beta$ bands, and the lack of one between RBP($\gamma$) and age.

The age model returned some singular fits at the regional level (Fig. 7B). These regions typically had a low number of data points and a small number of hospitals. We retained all models in all ROIs for completeness.

## Discussion

In this study, we considered how age, sex, and recording hospital, might impact RBP($\cdot$) in the setting of a normative icEEG map. To understand the effects of the variables, various LMMs were fitted, and it was concluded that sex and RBP($\cdot$) were not related in this cohort, whilst RBP($\cdot$) values present a moderate relationship with subject age and a notable relationship with the recording hospital.

This work provides insight into what the trajectory of RBP($\cdot$) extracted from icEEG might look like in healthy lifespan. Additionally, it confirms that hospital site effects must be considered when modelling multi-centre data, whilst also demonstrating that sex perhaps need not be considered when analysing normative icEEG RBP($\cdot$).

### The relationship between hospital, sex, age and band power

#### Hospital and band power

In any large-scale retrospective data analysis, there has to be a trade-off between variables we cannot control and maintaining a large sample size. We find it encouraging that, on average, similar normative maps are produced, indicating that the hospital effects aren’t dominating the data. However, we still observe substantial hospital-specific effects.

In other neuroscience research concerned with normative mapping, such as neuroimaging, the multi-centre problem is widely recognised (Hu et al. 2023; Keshavan et al. 2016; Jovicich et al. 2006). It is common practice to model hospital site effects (or ‘batch’ effects in neuroimaging literature) as random offsets in normative models (Little et al. 2024; Hibar et al. 2018; Ge et al. 2024; Bethlehem et al. 2022; Kim et al. 2022). Whilst it is impossible to capture all sources of hospital (or scanner) heterogeneity, there is some consideration of them in this field. Meanwhile, other neuroscience studies, such as those in EEG, do not typically consider hospital site effects.

For example, the RAM dataset involves nine hospitals and is regularly used in academic work to produce results in icEEG (Taylor et al. 2022; Wang et al. 2023; Nozari et al. 2023; Kozma et al. 2024; Goldstein et al. 2019; Das et al. 2022). Whilst the multi-centre challenge here has been recognised before by Miller (2019), to our knowledge, this and other studies have not statistically accounted for the multi-centre structure present.

Our work reveals that, in the relative band power properties, the size of the hospital effect is frequency band specific, accounting for up to 30% of the variation. This not only demonstrates the necessity of hospital effect consideration, but also raises the question of what drives the differences. Supplementary S8 considers the metadata we have available—electrode type, age range and originating cohort (RAM/other)—as possible contributors to recording hospital differences, but finds no systematic variation in these factors. The former lines up with previous literature: Frauscher et al. (2018a) did not find any change in spectral properties due to electrode type. Similarly, Alkawadri et al. (2024) found the spectral properties for depth and subdural electrodes within close proximity to each other to be highly correlated.

Other technical aspects, such as recording settings, electrode manufacturer, or electrode design could be involved. Unfortunately, such information is not available for this cohort. Sindhu et al. (2023) find that electrode size can impact icEEG power and amplitude, suggesting more information on electrode design and manufacture should be collected and investigated in future works.

Possible demographic factors influencing the hospital effect include race, geography and the hospital’s subject selection criteria. Our data did not contain information on subject ethnicity, however, this data should be collected in the future, to address the issue of past exclusionary practices in neuroscience (Ricard et al. 2022; Li et al. 2022). Previous research found race and marital status to be marginally associated with whether or not an individual proceeds to epilepsy surgery (Berg et al. 2003), so it is plausible such factors could also impact whether individuals undergo intracranial examination.

The implementation of the state of vigilance criteria also varied between recording hospitals. The recordings from RAM hospitals were obtained prior to a memory task, confirming subjects were awake. For the remaining hospitals, some collaborators sent us wake-state recordings, whilst others sent long-term recordings from which we extracted segments in the wake-state. In the former case, the methods used to determine the state of vigilance are unknown, which is both a limitation of our work and a potential source of hospital variation. Future multi-site work should strive to standardise the implementation of the wake-state criteria across hospitals.

Finally, we wish to highlight the dependency of the hospital site effect size on signal feature in our cohort (Table 1). For example, the hospital site effect was weakest in RBP($\alpha$). Investigating whether this is reproducible in other datasets is a further avenue of future research. There may also exist signal features which are more robust towards the effect of recording hospital.

In summary, although the multi-centre effect is under-explored in icEEG research, it is present in our work. Future work should improve on our own by collecting more metadata, to help identify what is driving the hospital site effect and to explain why different signal properties yield different results.

#### Age and band power

Moving to the fixed effects, our work produced convincing results in the $\delta$, $\alpha$ and $\beta$ bands when testing for a relationship between RBP($\cdot$) and age. Specifically, RBP($\delta$) decreased with subject age, while RBP($\alpha$) and RBP($\beta$) increased. More generally, we found an increase in RBP($\cdot$) with age in fast bands and a decrease in RBP($\cdot$) with age in slower bands.

These fixed effect results concur with previous literature on scalp EEG, although here the age range is reduced to children and adolescents, which found fast bands increased and the slow bands decreased in RBP($\cdot$) (Gasser et al. 1988; Clarke et al. 2001). Using the same age range, MEG power spectral density maps found an analogous relationship between power and age (Ott et al. 2021).

Looking at the full lifespan (7–84 years) using MEG, previous work found identical trends (a decrease in RBP($\delta$) and RBP($\theta$), and the converse for higher bands); however, the authors report slight changes around the sixth decade of life (Gómez et al. 2013). Since our maximum age is 18 years less at 66, this result still complements our findings. Further, a study using a small set of scalp electrodes across a large adult cohort found the same age trajectories as ours between 20–40 years, including the strongest decrease being in the $\delta$-band (Hashemi et al. 2016). However, they found that these effects diminished in older age ranges, which our choice of linear modelling does not facilitate.

Finally, there is evidence in the literature of a decrease in alpha band power with age—Tröndle et al. (2023) and Turner et al. (2023) report this across adulthood, and Whitford et al. (2007) in adolescence. However, these studies use absolute power rather than relative, in scalp rather than intracranial EEG, so this does not contradict our findings. Tröndle et al. (2023) also argue that the decrease has been overestimated due to a lack of consideration of the aperiodic component.

#### Sex and band power

Our work did not find any evidence for a sex-RBP($\cdot$) relationship in this cohort. In the literature however, Ott et al. (2021) report sex differences in MEG power spectral density maps for children and adolescents in $\delta$, $\beta$ and $\alpha$. They do not report a main effect of sex in $\theta$ and $\gamma$, which are the same bands in which we found no notable relationship for either fixed effect. Further, Hashemi et al. (2016) report significant sex differences in total band power with age in an adult cohort using scalp EEG.

Nevertheless, as with the evidence for the lifespan decrease in alpha power, both studies use a different, although related, feature and modality, which may facilitate the sex differences. Finding a difference in absolute power (Hashemi et al. 2016), but not relative power (our cohort) may suggest that the distribution of power is the same for men and women, but that the values differ. Alternatively, it is plausible that there is a genuine effect of sex on RBP($\cdot$) but that it is too small to detect in our cohort.

In summary, the age trajectories we have found are generally in line with previous literature in similar areas, but a conclusion on the presence of sex differences remains unclear.

### Existing normative mapping work and how it relates to the ageing patterns

Normative mapping is well-established in neuroscience research showing promising results in a range of modalities including MEG, scalp EEG and icEEG (Niso et al. 2019; Owen et al. 2023b; Janiukstyte et al. 2023; Taylor et al. 2022; Frauscher et al. 2018a; Betzel et al. 2019). Evidence for the validity of normative maps has been studied, along with their temporal stability (Rutherford et al. 2023; Wang et al. 2023; Janiukstyte et al. 2023). Generally, the patterns noted in neurophysiological normative maps complement each other, indicating their value. For example, many identify well-known trends such as $\alpha$ dominance in parietal and occipital regions (Taylor et al. 2022; Owen et al. 2023b; Janiukstyte et al. 2023; Frauscher et al. 2018a; Groppe et al. 2013; Bosch-Bayard et al. 2020b).

The majority of normative mapping studies outlined consider their maps from a static viewpoint and did not incorporate potential age effects. In this study, we demonstrated that RBP($\cdot$) varies with age and that this variation is frequency band-specific. This is not surprising, as there is much previous work evidencing that healthy ageing *does* impact brain activity in modalities such as MEG and scalp EEG (Gomez et al. 2017; Gómez et al. 2013; Duffy et al. 1993). Therefore we suggest, based on our findings, that future work no longer considers icEEG normative maps as a static snapshot and instead strives to incorporate dynamic features, such as age, into any study.

### Clinical potential

Turning now to an application of normative maps, the comparison of individuals with epilepsy (or other disorders) to normalised healthy controls is a common one. It is gaining traction in the field of neurophysiology, using modalities which facilitate said controls such as scalp EEG and MEG (Bosch-Bayard et al. 2020a; Owen et al. 2023b; Janiukstyte et al. 2023; Owen et al. 2023a). Similar work has been produced in the invasive icEEG setting; hence, it is plausible that the ageing trends demonstrated here could yield similar clinical potential (Taylor et al. 2022; Bernabei et al. 2022; Kozma et al. 2024).

Whilst readily interpretable plots such as those in Fig. 6B summarise findings well, the core analysis is LMMs fitted at regional (Figs. 6A) or whole-brain levels (Table 2). Therefore, to localise pathological tissue in a clinical setting following an icEEG exam, we might compare an individual’s age and RBP($\cdot$) in each frequency band to the corresponding regional regression line, quantifying differences, to determine whether their RBP($\cdot$) deviates from expected values at that age. Identifying regions with extreme deviations could guide the location of further examination. This particular pipeline would be similar to that suggested in Taylor et al. (2022). In the case of epilepsy, this could complement the standard seizure onset zone localisation techniques following an icEEG exam, without further work or procedures.

### Limitations and future directions

The concept of ‘normative’ data collected from individuals with epilepsy is perhaps contentious. These methods are defensible in neuroimaging (Rutherford et al. 2023) and have been applied in previous icEEG work (Bernabei et al. 2022; Frauscher et al. 2018a; Kalamangalam et al. 2020; Kozma et al. 2024; Taylor et al. 2022; Wang et al. 2023); however, epilepsy is increasingly defined as a network disorder (Rayner and Tailby 2017; Kramer and Cash 2012; Bernhardt et al. 2015), which contrasts with the notion of delineating normal and abnormal tissue. In particular, previous work has shown that more complete resection of seizure onset regions is not associated with more favourable surgical outcomes (Gascoigne et al. 2024). Hence, it is arguable that the icEEG description of pathological tissue is complex and incomplete.

Additionally, sample size impacted results despite our efforts in data collection (Sect. 3.5), with high regional variation in number of subjects (Fig. 3). In an ideal world, we would have a similar number of subjects per region, allowing for more accurate comparison. In practice, however, this is not feasible due to some areas being more prone to pathology and therefore being more likely to have electrodes implanted– for example, drug-resistant epilepsies in adults are common in the temporal lobe (Bernhardt et al. 2019).

A further drawback is that our maximum age was only 66 years, so we did not have the full lifespan. This will always be difficult due to the risk of surgical operations on the elderly (Grivas et al. 2006), which limits the ability to directly compare our results to the many other modalities which extend to much older ages (Gómez et al. 2013; Cam-CAN et al. 2014; Hashemi et al. 2016; Duffy et al. 1993). Arguably the minimum age of our cohort (4 years) also does not reflect the full lifespan, however, research has shown that children at younger ages have successfully undergone icEEG-guided epilepsy surgery and tolerated the invasive exam well (Taussig et al. 2016). Future data collection efforts could focus on expanding our age range at both ends.

Similarly, the proportion of paediatric individuals in our cohort is low (Fig. 1). In future work, we aim to collect more paediatric recordings and analyse this age range in isolation in an icEEG normative setting. This would provide insight into the effects of ageing during this key stage of life, as previously done for both scalp and MEG (Ott et al. 2021; Gomez et al. 2017; Gasser et al. 1988; Clarke et al. 2001).

Future work might also consider different frequency ranges. This study involves frequency content from 1$-$77.5 Hz, but higher frequency bands can contain pathological high-frequency oscillations (HFOs), which are thought to delineate epileptogenic tissue (Zweiphenning et al. 2016, 2019). Previous work has provided a normative map for HFOs obtained from icEEG (Frauscher et al. 2018b). Hence, accounting for HFOs as in Kuroda et al. (2021) and extending the frequency range of our normative map could improve its clinical potential for identifying pathological areas of the brain.

A further line of enquiry would be to increase the complexity of the model from *linear* mixed models and consider the quadratic case or beyond. This has been done in other modalities, which found some frequency bands required non-linear models of band power over age, whilst other bands did not (Gómez et al. 2013; Duffy et al. 1993). Future work might investigate whether icEEG trajectories mirror those results, or if perhaps the optimal model complexity is not only frequency band specific, but regionally specific as well.

Combining the discussions around frequency ranges and model complexity, there is also evidence that more granular frequency bands could affect the model selection process. For example, using healthy MEG data to determine how RBP($\cdot$) changes with age, Gómez et al. (2013) found that a linear regression model performed best for RBP(low-$\beta$), but a quadratic model is preferable for RBP(high-$\beta$). Analogous results were found in scalp EEG in the $\alpha$-band as well as the $\beta$-band (more complex models were required for the upper end of the band range) (Gasser et al. 1988).

Different features of EEG could also be considered, in particular, absolute band power as an alternative to the relative band power used here. This would allow for direct comparison to the aforementioned work which finds sex differences in absolute power (Hashemi et al. 2016). However, a previous EEG study investigating age, sex and band power found that absolute power failed to find significant lifespan effects in all bands except $\delta$, whilst relative power succeeded, suggesting further investigation using absolute power is unnecessary (Clarke et al. 2001). In future work, time-varying power could also be analysed. This has been done, for example, in a previous study which considers seizure severity (Gascoigne et al. 2023).

Similarly, different referencing systems could be considered. The choice of referencing system can impact icEEG recordings (Parish et al. 2023), and may therefore influence subsequent results. Some studies find the Laplacian reference to be optimal (Liu et al. 2021; Li et al. 2018), however these studies have a notably small sample size ($n=15$, $n=8$ respectively) and only consider icEEG recordings during motor tasks.

Finally, with our primary focus being the cohort’s size, we could not attain key metadata across all subjects, as discussed in Sects. 2.4 and 4.1.1. Outside of the aforementioned variables potentially driving the hospital effect (Sect. 4.1.1), ideally, subject-specific metadata would be considered, such as epilepsy type or medication dosage. In particular, anti-seizure medication tapering (which often occurs during icEEG monitoring to induce seizures) has been shown to affect band power (Zaveri et al. 2010; Besné et al. 2025). Such data should therefore be collected and analysed, if possible, in future works.

### Conclusions

In conclusion, our results suggest that whilst recording hospital and age have some impact on normative icEEG RBP($\cdot$) in this cohort, sex does not. Our work might be considered the first attempt to study the relationship between these variables and icEEG in a normative setting. Our results highlight the importance of accounting for the heterogeneity in icEEG data by including covariates such as age and recording hospital (where applicable) in future normative mapping work.

Sample size is a key discussion point of this study, being both a strength and limitation of our work. Whilst we have collected one of the largest icEEG datasets to date, some of our results are limited by number of subjects per region. Future work could strive to address this, whilst also considering the impact of non-linear models, or the effects of using a different EEG feature.

We propose that the dynamic nature of normative mapping should be acknowledged in future icEEG research, rather than only considering a static viewpoint that does not account for variables such as age. Further, multi-centre work needs to investigate and model the impact of using data from several recording hospitals to ensure the accurate interpretation of any results.

## Supplementary Information

Below is the link to the electronic supplementary material.

Supplementary file 1 (pdf 6168 KB)

## Data Availability

The preprocessed, normative data table including RBP($\cdot$) values, along with code used for modelling and to produce figures, will be made available at https://github.com/H-Woodhouse/norm-ieeg-age-sex-hospital upon acceptance. The data from RAM hospitals is publicly available at https://memory.psych.upenn.edu/RAM. Due to data sharing agreements, the raw icEEG for the remaining hospitals is not available. However, the CNNP lab will be publishing a data release paper containing this in the future.
