# Supplementary material for: Multi-centre normative brain mapping of intracranial EEG lifespan patterns in the human brain
Source: Brain Struct Funct. 2025 Aug 21;230(7):138. doi: 10.1007/s00429-025-02988-4 (PMC12370820; doi:10.1007/s00429-025-02988-4)
Supplement: Supplementary file 1 — (pdf 6168 KB) [file 429_2025_2988_MOESM1_ESM.pdf]

Multi-centre normative brain mapping of intracranial EEG lifespan patterns in the human brain

Journal: Brain Structure and Function

Heather Woodhouse[1], Gerard Hall[1], Callum Simpson[1], Csaba Kozma[1], Frances Turner[1], Gabrielle M. Schroeder[1], Beate Diehl[3], John S. Duncan[3], Jiajie Mo[4], Kai Zhang[4], Aswin Chari[5], Martin Tisdall[5], Friederike Moeller[5], Chris Petkov[2,6], Matthew A. Howard[6], George M. Ibrahim[7], Elizabeth Donner[7], Nebras M. Warsi[7], Raheel Ahmed[8], Peter N. Taylor[1,2,3], Yujiang Wang[1,2,3*]

1. CNNP Lab (www.cnnp-lab.com), Interdisciplinary Computing and Complex BioSystems Group, School of Computing, Newcastle University, Newcastle upon Tyne, United Kingdom
2. Faculty of Medical Sciences, Newcastle University, Newcastle upon Tyne, United Kingdom
3. UCL Queen Square Institute of Neurology, Queen Square, London, United Kingdom
4. Beijing Tiantan Hospital, Beijing, China
5. Great Ormond Street Hospital for Children, London, United Kingdom
6. University of Iowa Hospitals and Clinics, Iowa City, IA, United States
7. The Hospital for Sick Children, University of Toronto, Toronto, Canada
8. University of Wisconsin-Madison, Madison, WI, United States

* Yujiang.Wang@newcastle.ac.uk

Supplementary Material

S1 Algorithmic detection of noisy or faulty channels

Channels were removed based on the five criteria given below. American hospitals were run separately from the European hospitals and Beijing, due to the line noise difference.

1. Exclude channels based on channel details data. If channels are known to be resected, within structurally abnormal tissue or within the seizure onset zone, they are removed. This is determined by resection masks or clinical reports.

2. Exclude algorithmically detected noisy channels by finding channels with outlier signal range and/or variance relative to the other channels.

   - Two rounds of detection are performed; the first round is before preprocessing with (by default) less stringent detection thresholds, and the second is after basic preprocessing.
   - The first round uses a threshold for outlier detection of 16, for both signal variance and range.
   - Before the second round, common average is applied to the icEEG data.
   - A 4th order band pass filter is applied between 0 and 100 Hz.
   - A notch filter is applied to eliminate the location-specific line noise (50 Hz for European hospitals and Beijing Tiatian Hospital and 60 Hz for American hospitals).
   - The threshold for outlier detection based on signal range and variance in the second round following preprocessing is 12.

3. Exclude channels previously marked as ‘bad’. These are the channels noted as noisy/faulty by visual inspection.

4. Remove channels which are missing channel details.

5. Remove channels without mapping/localisation to an ROI.

S2 Available subject details by hospital site

Although age and sex information was available for all subjects, other variables such as the individual's age at the time of epilepsy onset were not. Table S1 shows the number of subjects per hospital that had information available for a range of clinical variables. This provides further insight into the condition of the normative data and verifies that these features could not be investigated without compromising the size of the dataset – a key advantage of this study.

Separately from clinical variables, three additional channel features were recorded, if available, through reports or resection masks, namely: whether the channel was 1) within the seizure onset zone, if determined; 2) located in structurally abnormal tissues, such as lesions; or 3) resected during surgery. If a channel fell into any of these categories, it was removed from analysis. Table S1 demonstrates how many subjects (per hospital site) had these variables in their reports.

	Number of subjects	Number of subjects with information on:					
		Age of epilepsy onset	Treatment type	Treatment outcome	SOZ	Structural abnormality	Resection
Beijing Tiantan Hosp.	32	32	26	26	32	0	28
Columbia University*	1	0	0	0	1	1	0
Dartmouth University*	26	0	0	0	26	26	0
Emory University*	34	0	0	0	34	34	0
Great Ormond St. Hosp.	15	15	14	14	15	0	13
Jefferson Hosp.*	41	0	0	0	41	41	0
Mayo Clinic*	35	0	0	0	35	35	0
NINDS*	16	0	0	0	16	16	0
SickKids	28	28	0	0	0	0	0
University of Pennsylvania*	29	0	0	0	29	29	0
University College London Hosp.	107	107	107	107	102	0	106
University of Iowa Hosp.	84	43	50	50	22	0	51
University of Washington*	2	0	0	0	2	2	0
University of Wisconsin-Madison	21	21	18	18	21	0	0
UT Southwestern*	31	0	0	0	31	31	0

Table S1: Overview of available data. The table comprises the number of subjects per hospital, and how many of those subjects have information on: age of epilepsy onset, the type of treatment (e.g., resective surgery) and the treatment outcome (either ILAE or Engel scores). For each hospital site, the number of subjects with information on which electrode contacts are within SOZs, within structural abnormalities or resected, is also reported. Hospitals from the RAM cohort are highlighted with an *.

S3 Robustness to parcellation choice

Our main results use the coarsest scale parcellation, scale 36, with data mirrored across hemispheres for sample size reasons. Here, we repeat Figure 6B again in a finer-grained parcellation, the scale 60 atlas. Here, the data has *not* been mirrored.

Due to a decrease in sample size per region, more regions produce a singular fit. Using the scale 36 atlas, as shown in Figure 7B, there are a total of 9 singular fits out of 190 total fits (38 ROIs $\times$ 5 bands). When using the scale 60 atlas, without mirroring, there are 61 singular fits out of 610 total fits (122 ROIs $\times$ 5 bands), which is a significantly larger portion – approximately double. This is due to the LMM fitted in each ROI being too complex for the size of the data at this finer-grained parcellation. For this reason, we did not consider any finer-grained atlases.

Nevertheless, as shown in Figure S1, using the scale 60 atlas, results are reasonably symmetric in δ, α and β. Any deviations from symmetry/expected results, tend to lie near the midline, whose regions have lower sample sizes than lateral ones. The θ and γ bands do not exhibit symmetry, but as outlined in Section 3.3, their relationship with age was weak.

We conclude that while (for this cohort) the model under consideration is only suitable for the parcellation applied in the main paper, symmetry holds to a satisfactory level when considering one, finer-grained parcellation.

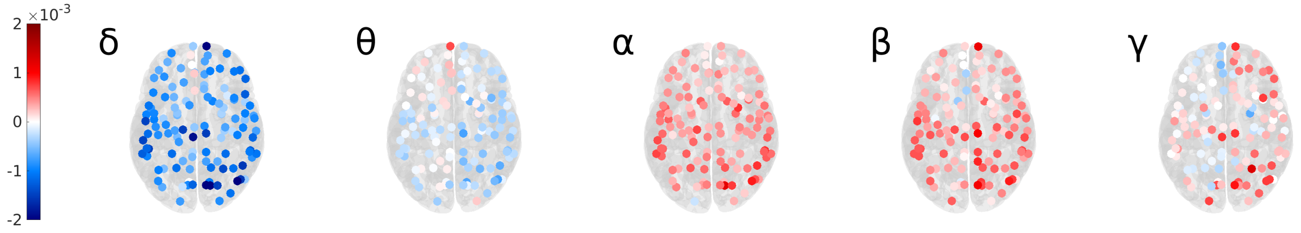

Figure S1: Values of $\hat{b}_{age}$ from the age model implemented at the region-level for the scale 60 atlas. Values are shown for each ROI and each frequency band of interest. The colour scale is symmetric and fixed across frequency bands with blue representing negative regression coefficients and red representing positive ones. The original data has been used so results are not reflected across the midline.

S4 Validity of mirroring data in our parcellation

In Section 2.4 we described the mirroring of symmetric regions in order to increase the sample size in each region. The only instance where we mirror across the midline for visualisation is found in Figure 6B. Here we repeat that figure, without mirroring symmetric regions, to demonstrate that the main results are unchanged. Note that, due to the reduction in sample size per ROI using the full data, there are 28 singular fits, treble the number attained when using mirrored data.

Figure S2 demonstrates that results are symmetric when using the original data in bands where we have identified a relationship between RBP($\cdot$) and age, and the overarching trends are unchanged. We see a decrease in RBP(δ) and RBP(θ) with age, an increase in RBP(α) and RBP(β), and weak results for RBP(γ). There is no spatial specificity in the results, and trends are weaker in the θ and γ bands. This is all in line with results presented in Result 3.4.

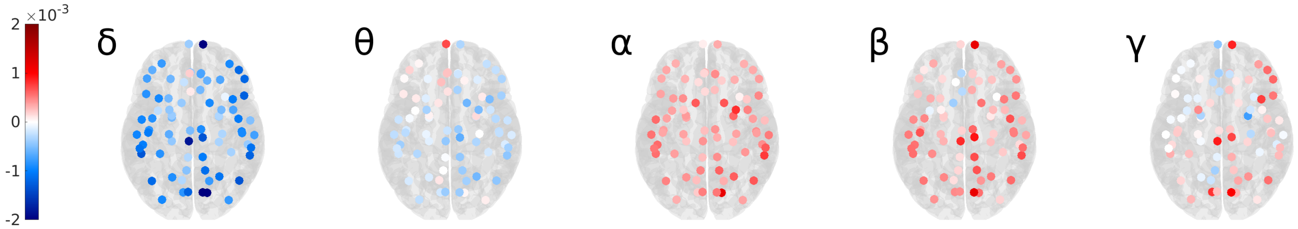

Figure S2: Values of $\hat{b}_{age}$ from the age model implemented at the region-level for the scale 36 atlas. Values are shown for each ROI and each frequency band of interest. The colour scale is symmetric and fixed across frequency bands with blue representing negative regression coefficients and red representing positive ones. The original data has been used so results are not reflected across the midline.

S5 Normative values are not correlated with age of epilepsy onset

After implementing the age model in all frequency bands at the ROI level using the mirrored data, we tested for correlation between model residuals and age of epilepsy onset.

Not every subject has age of onset data, so we fit the age model to all data, then applied Spearman's test using only complete pairs. Figure S3 shows the absolute value of the correlation estimate in each ROI and frequency band (top). Near-white values demonstrate strong correlation (in either direction) whilst dark red values indicate no, or weak, correlation. The p-value plot

(bottom) on the same axes shows a dark orange when $p < 0.05$. It shows a light orange if the converse is true. The x-axis is ordered from lowest to highest number of subjects per region.

Figure S3 is dominated by low correlations and non-significant p-values, indicating no relationship between age of onset and normative band power values, and further supporting that our data are representative of normative, rather than pathological activity.

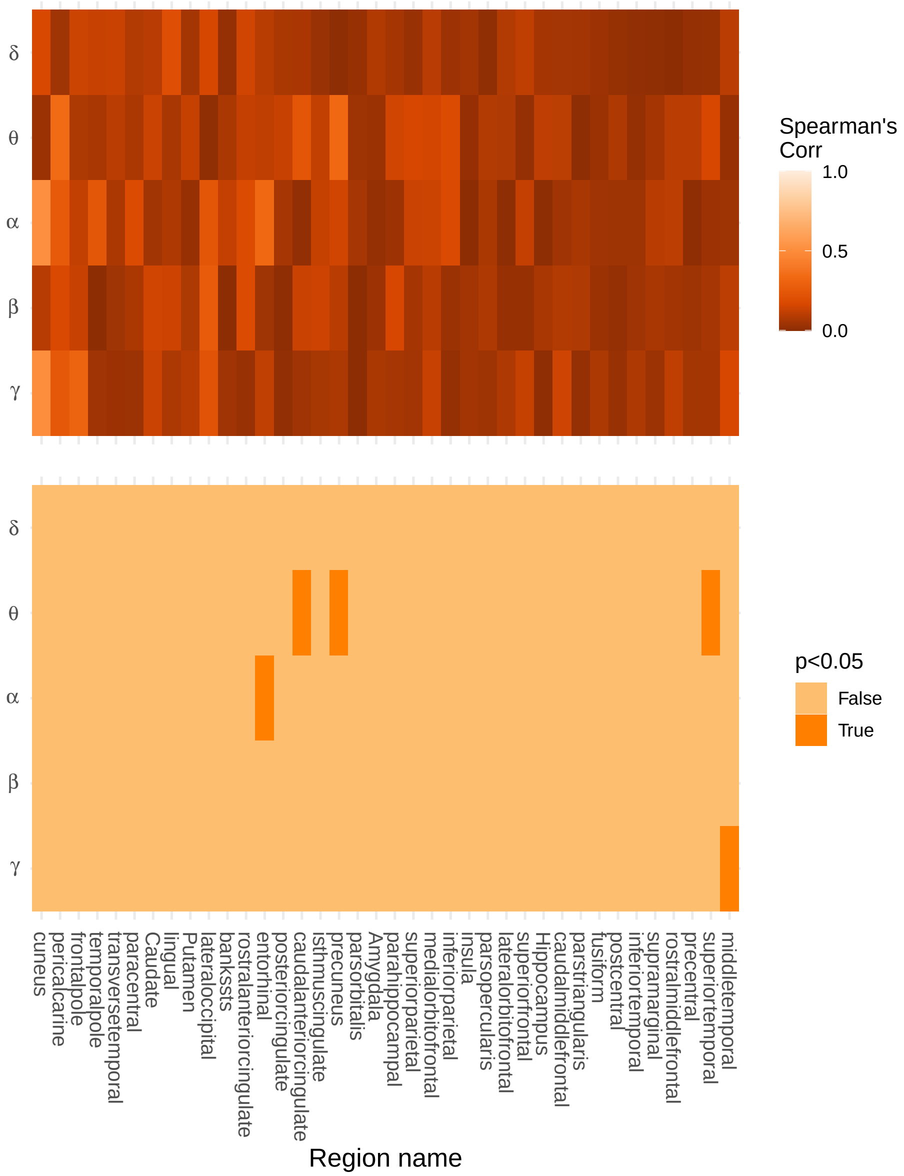

Figure S3: Heatmaps representing Spearman's correlation test applied to the residuals of the age model and age of epilepsy onset, in every frequency band and ROI. Absolute correlation coefficients (top) are on a gradient colour scale with near-white representing high correlation and dark red representing low values. Corresponding p-values (bottom) have a binary indicator of significance at the 5% level. We have not applied a multiple-comparison correction, but the rate of detection is in agreement with and below what would be expected at 5% level (5 detections out of 190 tests). The regions are ordered by number of subjects, from low to high.

S6 No evidence of a non-linear relationship between RBP(·) and age

We decided a linear mixed model would be suitable for this work (Section 2.5) as we did not see strong evidence that the relationship between RBP(·) and age was non-linear. Figure S4 shows all data at the whole-brain level, and presents a generally linear trend in each frequency band.

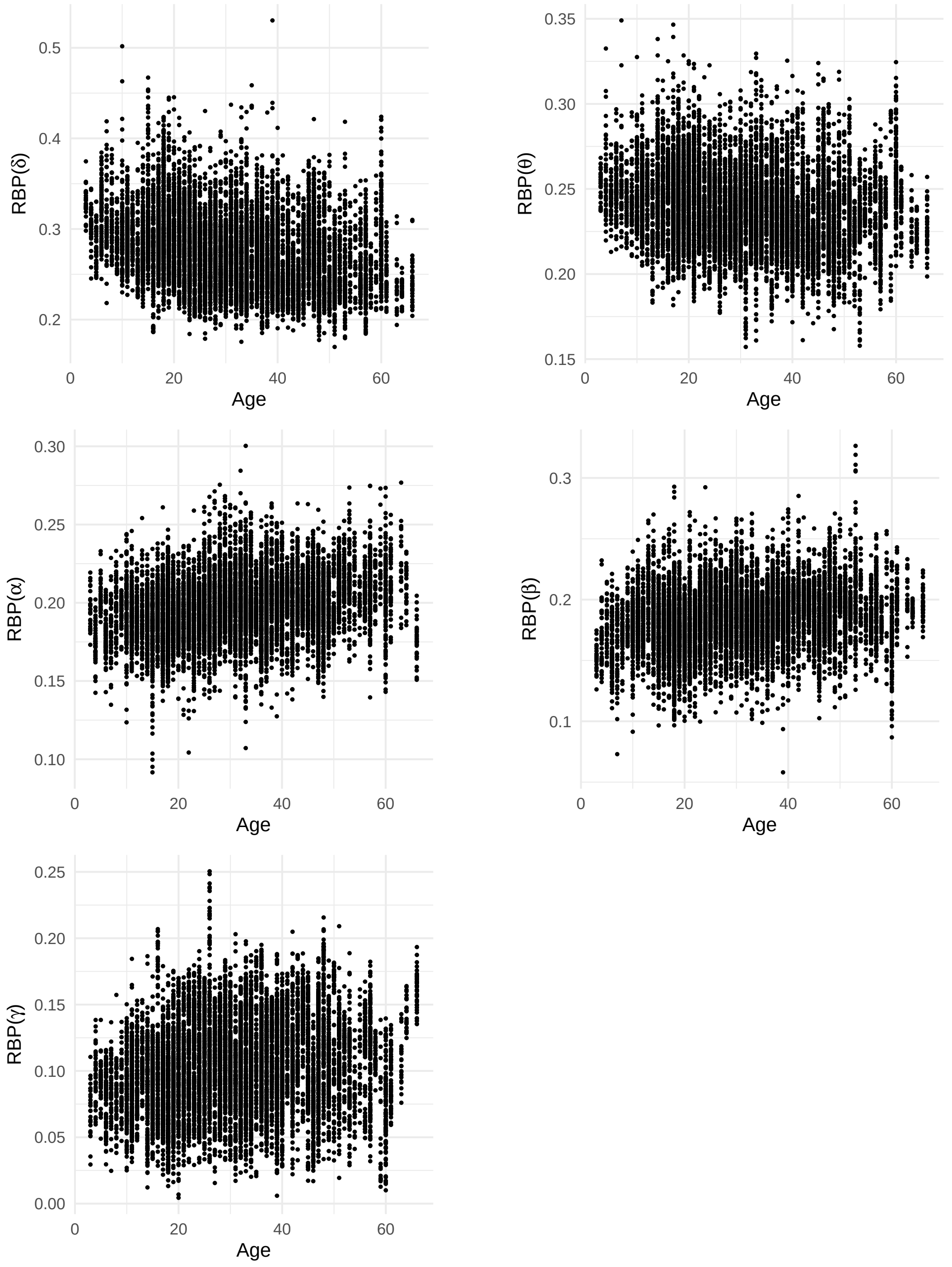

Figure S4: Scatter plots of RBP(·) against age in each frequency band, using the original data at the whole-brain level

S7 Consideration of an interaction term between age and sex

During the model selection process in Section 2.5, we additionally considered a fifth covariate structure involving an interaction term, giving a final model for each frequency band, $RBP(\cdot) \sim Age + Sex + Age * Sex + (1|Hospital)$.

During the model selection process, δ, β and γ returned non-zero confidence intervals on the interaction term, however, model evaluation statistics did not unanimously select the interaction model, and it performed similarly to the simpler, optimal choice in each case. In the α-band the interaction was not significant. The θ-band selected the interaction model, however as determined throughout our results, RBP(θ) has a weak relationship with our covariates. For these reasons, we didn't consider the interaction term in the main text.

To demonstrate the lack of value of an interaction term further down the pipeline, we repeat Table 1 here with the interaction model included. Looking at the R_m^2 columns, the inclusion of an interaction term adds a maximum of 0.18% to the variation in RBP($\cdot$) explained by our fixed effects structure. In the θ-band where the interaction model is preferred, the response variance explained by fixed effects is $< 1\%$. Hence an interaction term was not considered within our main results.

	Interaction model		Full model		Age model		Sex model	
	R_m^2	ICC	R_m^2	ICC	R_m^2	ICC	R_m^2	ICC
δ	4.46	15.26	4.28	15.23	4.27	15.27	0.00	19.42
θ	0.70	15.10	0.60	15.17	0.23	14.88	0.41	16.25
α	8.07	5.16	8.04	5.09	7.97	5.13	0.00	5.88
β	2.71	6.59	2.59	6.58	2.58	6.58	0.00	7.93
γ	0.37	32.55	0.25	32.59	0.01	32.16	0.24	32.25

Table S2: R_m^2 and ICC values (measured in %) for the interaction model, the full model, the age model and the sex model. R_m^2 represents the proportion of variation in RBP($\cdot$) explained by the fixed effect(s) of that model. The ICC represents the proportion of the variance explained by the grouping structure, namely recording hospitals. The optimal covariate subset, as determined by a standard model selection process, is highlighted for each frequency band.

S8 Hospital site effects across all frequency bands and hospitals

In Figure 4, we used one frequency band and three hospitals to demonstrate the hospital effect visually, overlaid on the normative data points. Here, we fit the age model on the whole brain level to demonstrate the varying hospital intercepts across all hospital sites and frequency bands, to highlight the importance of the consideration of such effects.

Figure S5 shows (for each frequency band) the deviation of each hospital's intercept from the population intercept, along with a standard 95% confidence interval. In other words, each hospital's deviation from the mean population RBP($\cdot$). Additionally, metadata has been included on the left of the plot, indicating which hospitals originated from the RAM database, which hospitals used only depth electrodes, and which hospitals had only paediatric, or only adult subjects. On the y-axis, hospitals are ordered by deviation from the overarching intercept in that band. The x-axis range is fixed across figures, demonstrating how some frequency bands show greater variation across recording hospitals.

Columbia University and the University of Washington consistently display notably large confidence intervals as a result of their low sample size. Whilst some hospitals fall above or below population means in each band, there are no hospitals which are systematically different across all five subplots. Further, there are no groupings, such as only paediatric hospitals, which show a consistent pattern across signal properties.

Hence, we do not have sufficient metadata to determine what is driving the large, varied hospital site effects, it does not appear to be any of the variables present in our cohort. Further investigation is required to determine the source of the recording hospital differences in RBP($\cdot$).

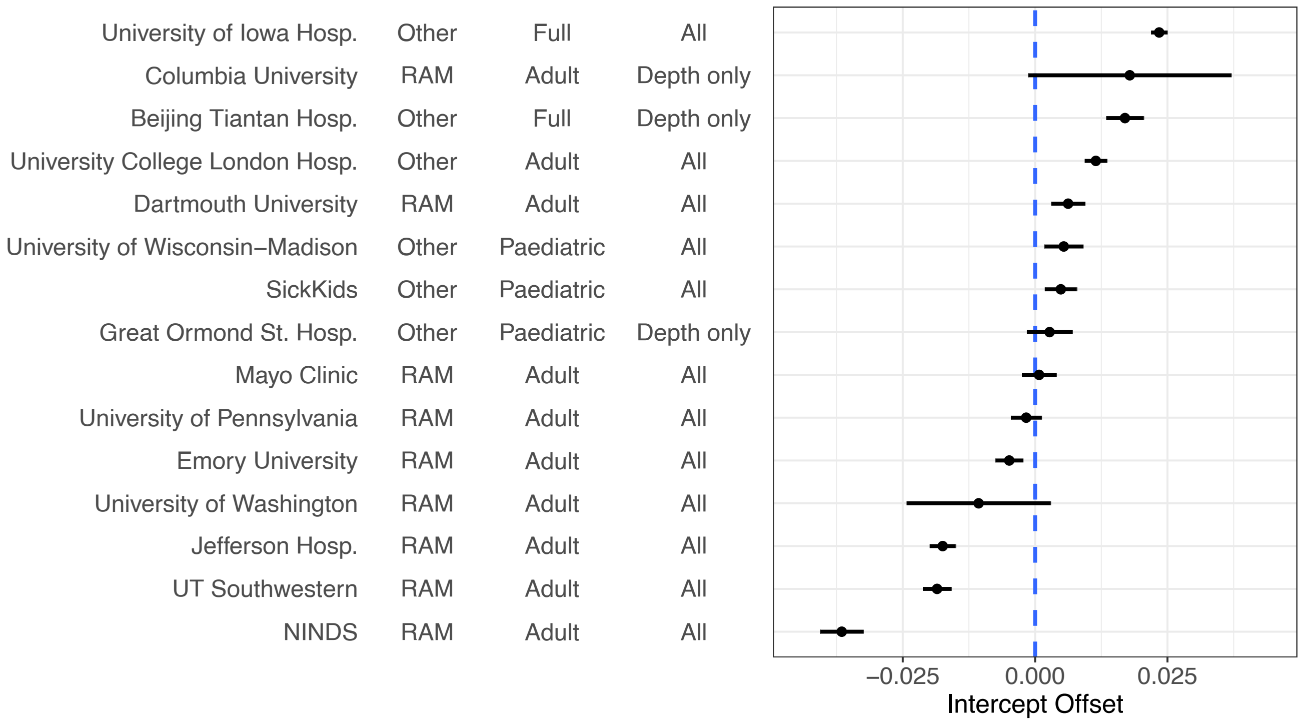

(a) δ-band

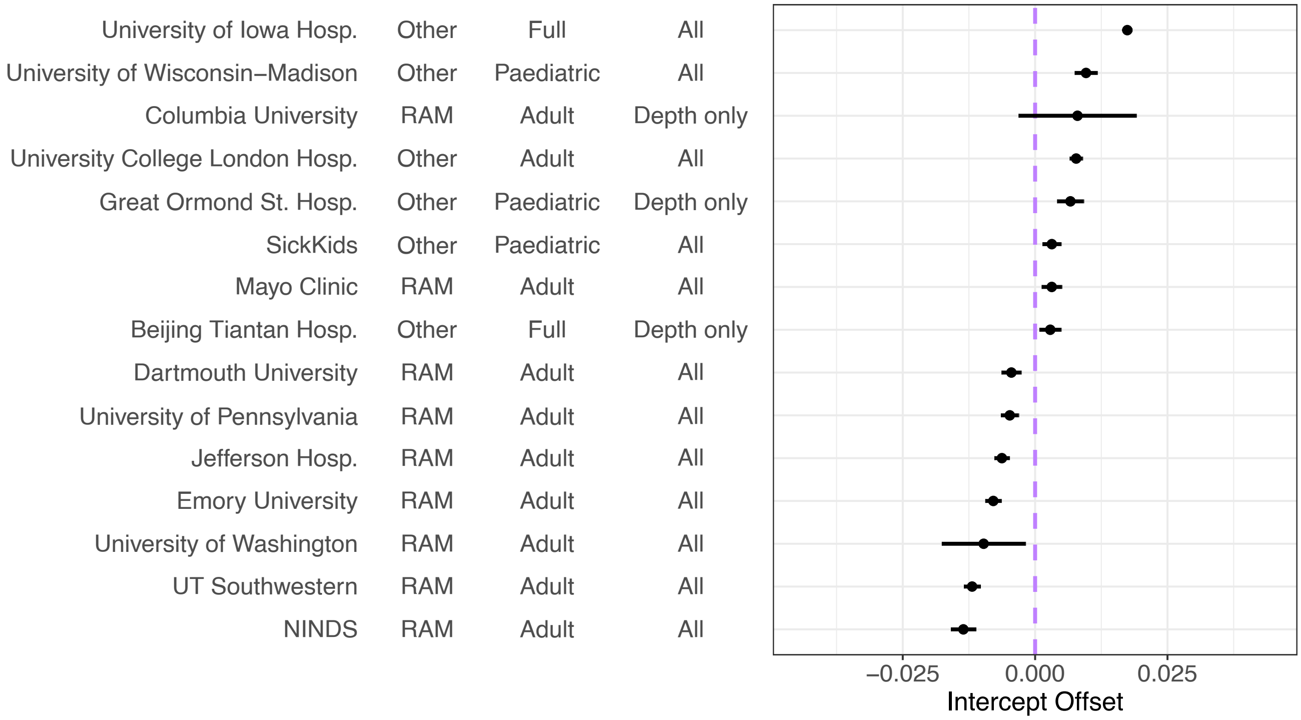

(b) θ-band

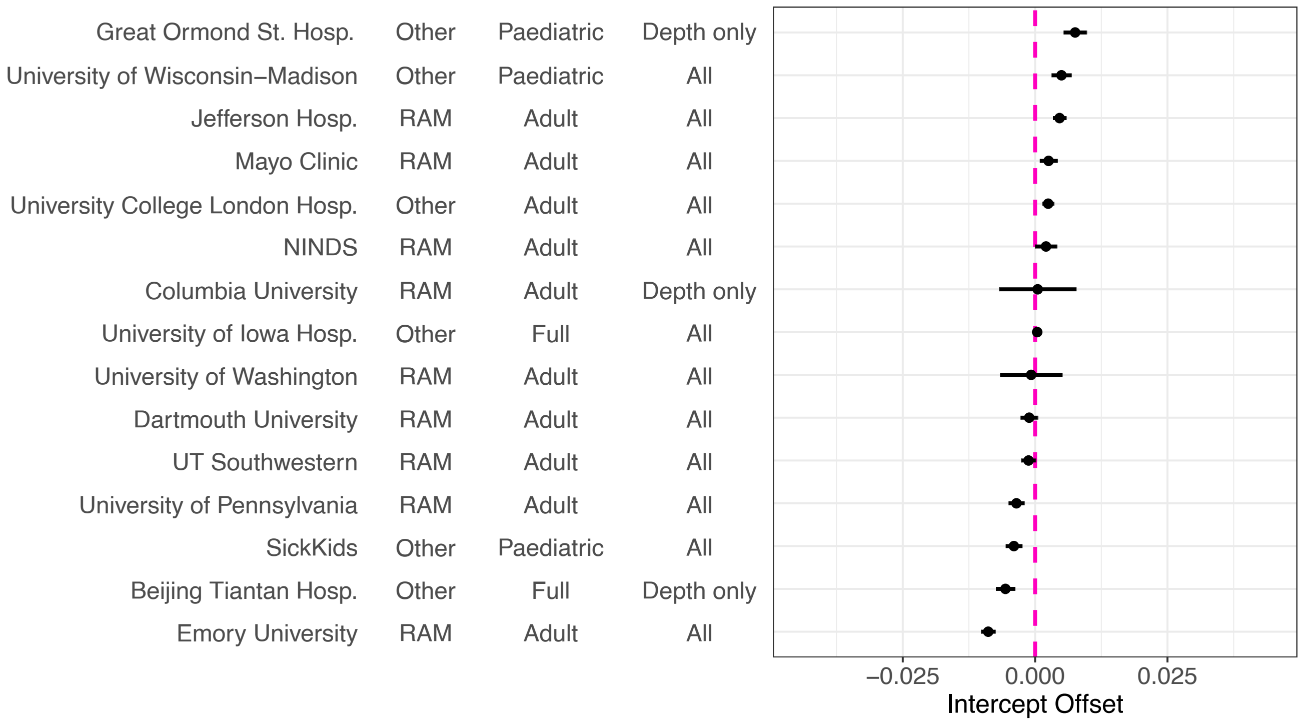

(c) α-band

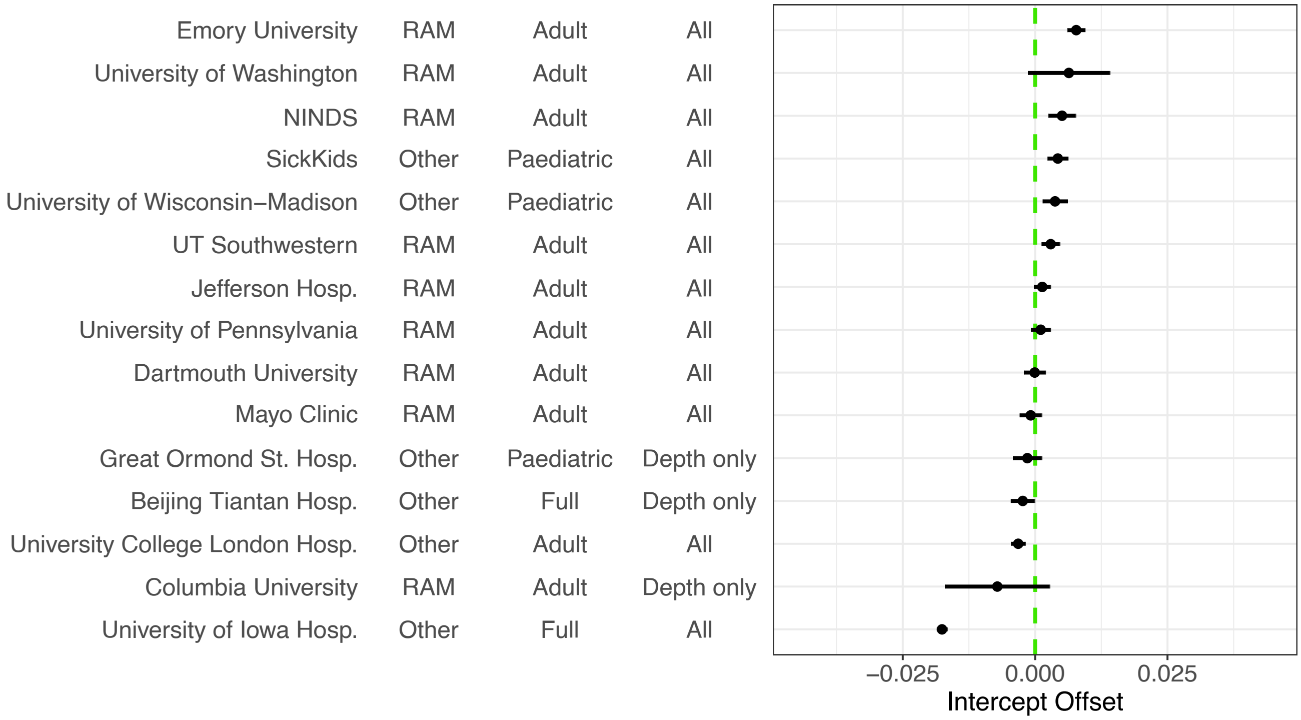

(d) β-band

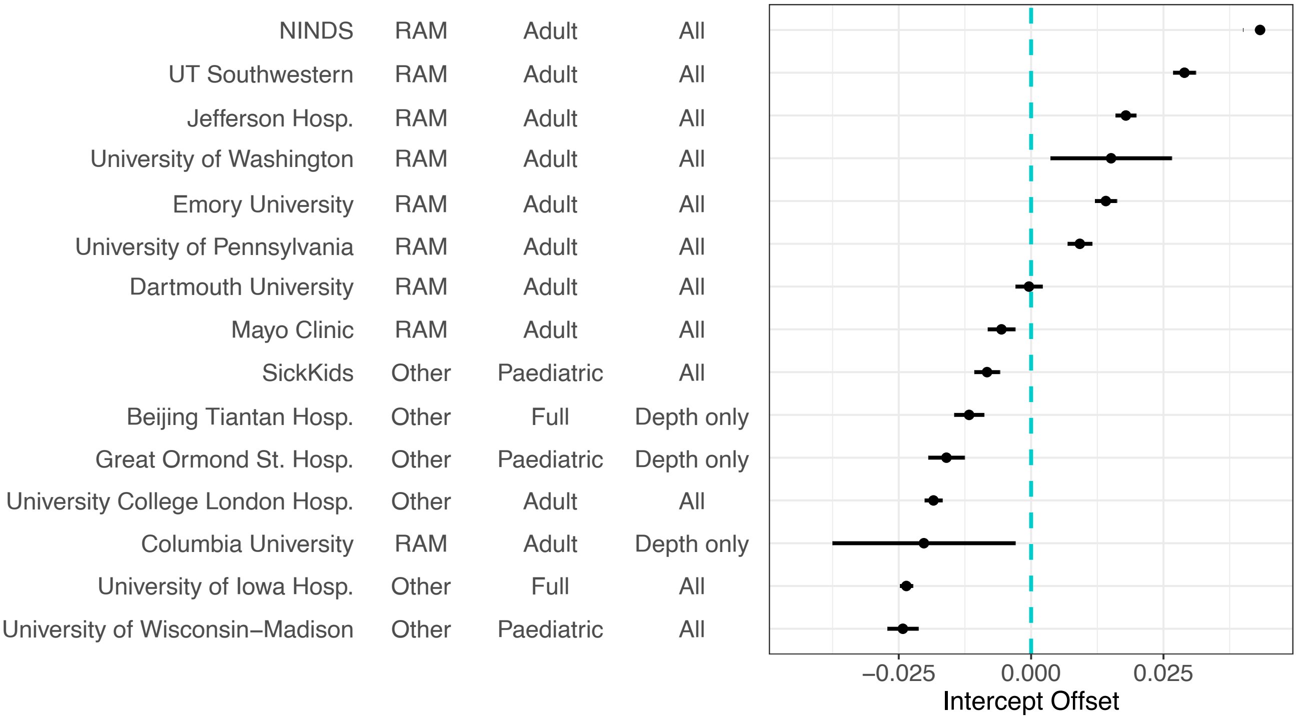

(e) γ-band

Figure S5: For each frequency band **a)-e)**: Plots showing each recording hospital's deviation from the population intercept when fitting the age model at the whole brain level. The x-axis is fixed at a range of $(-0.05, 0.05)$ for all bands. The hospitals are ordered on the y-axis by largest positive offset from the $\mathrm{RBP}(\cdot)$ population mean, to largest negative offset. Three metadata columns on the left of the plot show three grouping variables. Namely 1) if the hospital originated from the RAM dataset, 2) if the hospital comprises only paediatric subjects, only adults, or the full age range, 3) if the retained subjects from the hospital had only depth electrode implantation, or whether there were some individuals with grid/strip electrodes as well.

S9 A more detailed look at icEEG segments for three example patients

In Figure 4 we provided icEEG traces for three subjects from three different hospitals of the same age and sex (33 years old, male). This aided in demonstrating that although icEEG data may appear similar, the modelling process reveals an underlying effect of recording hospital. Here, we consider the icEEG segments of the same three subjects in more detail.

Figure S6 shows two 5-second snippets taken from each subject's 70-second preprocessed, interictal icEEG segment. Each snippet shows a different subset of channels originating from the same lobe. All three subjects had frontal lobe implantation, while two also had parietal lobe implantation, allowing for comparison across subjects. The individual examined at Mayo Clinic did

not have sufficient channels implanted in the parietal lobe, but they did have significant temporal lobe implantation, which the other two subjects did not. Hence, we include some of their temporal lobe channels for completeness. In each row of the figure, we intentionally keep the number of channels the same (to match the subject with the least available) to facilitate comparison.

Figure S6 further demonstrates that even when examining a narrower time frame, hospital differences are not visually apparent. This figure also demonstrates the quality of a typical icEEG segment from our dataset.

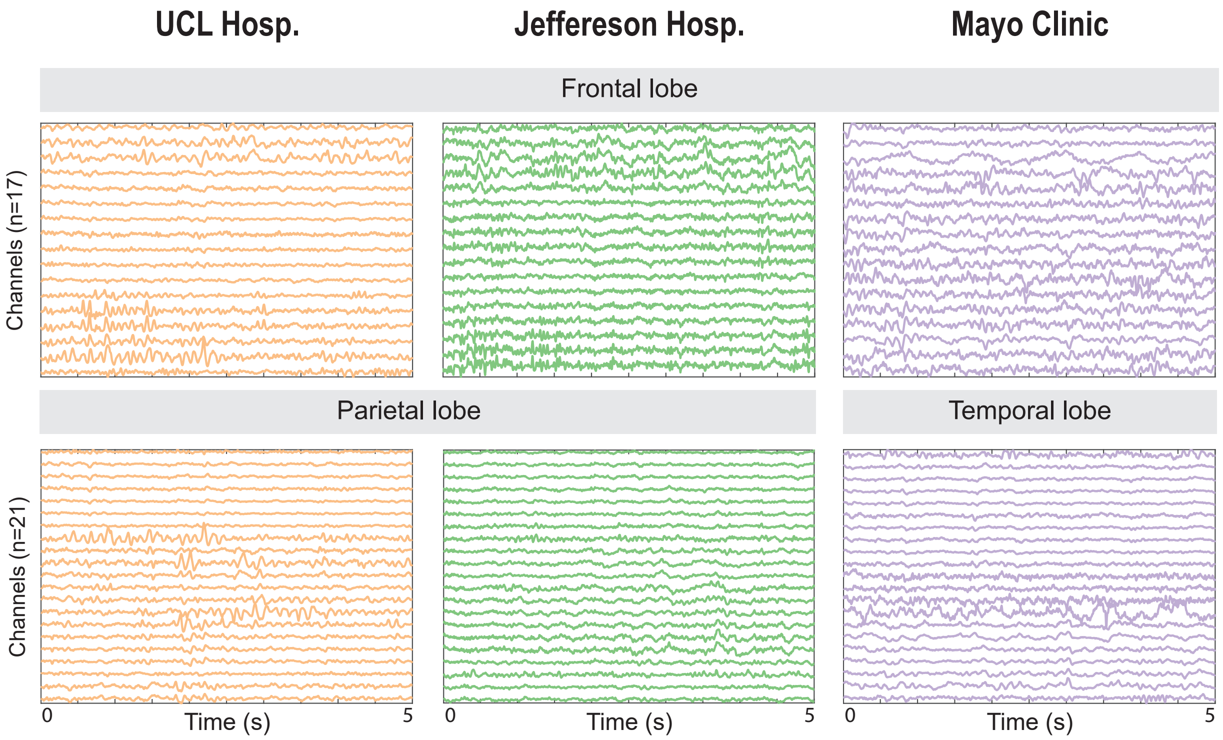

Figure S6: Two example icEEG segments with differing channel subsets for three men aged 33. Each subject was recorded at a different hospital (University College London Hospital, Jefferson Hospital and Mayo Clinic). For each segment, the first 5 seconds were extracted from their preprocessed, 70-second interictal segment. In each case, channels were grouped by lobe as demonstrated by the grey boxes.

S10 Age distributions at ROI-level

Results 3.4 and 3.5, employ the age model at the regional level. Whilst we discuss the number of subjects per model, this could be misleading in this context if, for example, a highly populated ROI only consisted of a very narrow age range. Such a scenario would undoubtedly impact any

results surrounding $\hat{b}_{age}$.

Hence, we have calculated the 2.5th and 97.5th percentiles to give the 95% central age range in each region, and plotted this against the regions $\hat{b}_{age}$ value. This has been repeated for all five frequency bands and the result can be seen in Figure S7.

All ROIs have a minimum 2.5-97.5th percentile age range of 43 years, with about half the regions reaching 50 years or more. Additionally, in all five frequency bands, there is no correlation between $\hat{b}_{age}$ and the distribution of ages, as confirmed by Spearman's method. Therefore, there is a reasonable distribution of subject ages in each ROI, and there is no evidence that this distribution is influencing regional results. It is acceptable to discuss sample size of regions as the influencing factor in the relevant results.

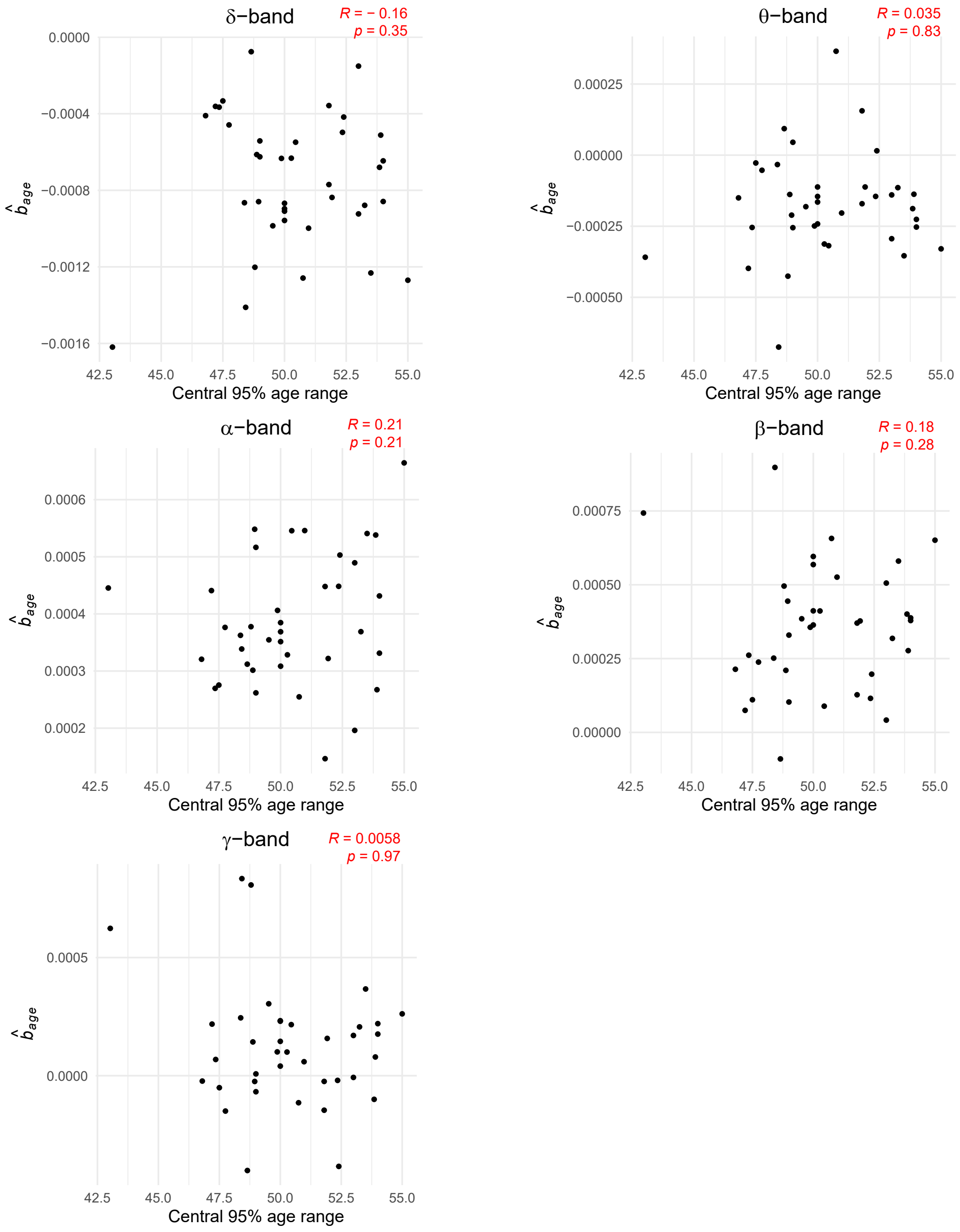

Figure S7: Values of $\hat{b}_{age}$ from the age model implemented at the region-level for the scale 36 atlas plotted against the central 95% percentile range of subject ages in that region. Results are given for all frequency bands with Spearman's correlation coefficient and resulting p-value in the top-right corner.
